# Jagged 1 is a major Notch ligand along cholangiocarcinoma development in mice and humans

**DOI:** 10.1038/oncsis.2016.73

**Published:** 2016-12-05

**Authors:** L Che, B Fan, M G Pilo, Z Xu, Y Liu, A Cigliano, A Cossu, G Palmieri, R M Pascale, A Porcu, G Vidili, M Serra, F Dombrowski, S Ribback, D F Calvisi, X Chen

**Affiliations:** 1 Key Laboratory of Carcinogenesis and Translational Research (Ministry of Education), Peking University Cancer Hospital & Institute, Beijing, China; 2 Department of Bioengineering and Therapeutic Sciences and Liver Center, University of California, San Francisco, CA, USA; 3 Institute of Pathology, University of Greifswald, Greifswald, Germany; 4 Department of Gastroenterology, Guizhou Provincial People's Hospital, The Affiliated People's Hospital of Guizhou Medical University, Guiyang, China; 5 Department of Gastroenterology, 307 Hospital of PLA, Beijing, China; 6 Unit of Pathology, Azienda Ospedaliero Universitaria Sassari, Sassari, Italy; 7 Institute of Biomolecular Chemistry, National Research Council, Sassari, Italy; 8 Department of Clinical and Experimental Medicine, University of Sassari, Sassari, Italy

## Abstract

Intrahepatic cholangiocarcinoma (ICC) is a rare yet deadly malignancy with limited treatment options. Activation of the Notch signalling cascade has been implicated in cholangiocarcinogenesis. However, while several studies focused on the Notch receptors required for ICC development, little is known about the upstream inducers responsible for their activation. Here, we show that the Jagged 1 (Jag1) ligand is almost ubiquitously upregulated in human ICC samples when compared with corresponding non-tumorous counterparts. Furthermore, we found that while overexpression of Jag1 alone does not lead to liver tumour development, overexpression of Jag1 synergizes with activated AKT signalling to promote liver carcinogenesis in AKT/Jag1 mice. Histologically, tumours consisted exclusively of ICC, with hepatocellular tumours not occurring in AKT/Jag1 mice. Furthermore, tumours from AKT/Jag1 mice exhibited extensive desmoplastic reaction, an important feature of human ICC. At the molecular level, we found that both AKT/mTOR and Notch cascades are activated in AKT/Jag1 ICC tissues, and that the Notch signalling is necessary for ICC development in AKT/Jag1 mice. In human ICC cell lines, silencing of Jag1 via specific small interfering RNA reduces proliferation and increases apoptosis. Finally, combined inhibition of AKT and Notch pathways is highly detrimental for the *in vitro* growth of ICC cell lines. In summary, our study demonstrates that Jag1 is an important upstream inducer of the Notch signalling in human and mouse ICC. Targeting Jag1 might represent a novel therapeutic strategy for the treatment of this deadly disease.

## Introduction

Intrahepatic cholangiocarcinoma (ICC) is the second most common primary liver tumour, and is characterized by an extremely unfavourable prognosis.^1, 2^ In the past decades, the incidence of ICC has been increasing by threefold to fivefold worldwide, including the USA.^3, 4^ Treatment options for ICC are very limited.^5, 6^ While surgical resection can be applied to a minority of patients,^7^ chemotherapy is the standard care for advanced ICC. However, it has only very limited efficacy.^5^ Currently, there is no targeted therapy available for this tumour type,^6^ largely due to the fact that molecular mechanisms underlying ICC development and progression remain poorly defined.^8^ Importantly, recent genomic studies have identified novel pathways and mutations in ICC, such as FGFR signalling and IDH1/2 mutations, which could be eventually targeted with specific drugs.^9^ However, the contribution of these genetic and signalling events in cholangiocarcinogenesis requires further validation using *in vitro* and *in vivo* approaches.

Recently, the Notch cascade has emerged as a major signalling pathway promoting intrahepatic cholangiocarcinogenesis.^10, 11^ Notch is an evolutionally conserved pathway that regulates development, stem cell function, angiogenesis and carcinogenesis.^12, 13, 14^ Notch signalling is unique in that both Notch receptors (Notch1, 2, 3 and 4) and ligands (Jag1, Jag2, DLL1, DLL3 and DLL4) are transmembrane proteins and the activation of Notch signalling requires direct cell-cell contact.^15^ Once induced, Notch receptors are cleaved from the membrane, allowing the translocation of the Notch intracellular domain (NICD) into the nucleus, where it binds to the recombination signal binding protein for immunoglobulin kappa J (RBP-J) transcription factor and activates downstream effectors, including Hes1, Hes5 and Hey1.^12, 15^ Notch signalling plays a crucial oncogenic role in several tumour types^13^ and targeting the Notch cascade might represent a valuable strategy for cancer treatment.^16^ In the liver, the Notch pathway contributes to hepatic development, and is required for biliary differentiation.^10, 17, 18^ Of note, mutations of genes belonging to the Notch pathway, including *Jag1* and *Notch2*, have been identified in patients with Alagille syndrome, a human genetic disorder characterized by paucity of intrahepatic bile ducts, leading to chronic cholestasis and liver failure.^19^ In human ICC, the Notch pathway is frequently activated.^20, 21^ Importantly, overexpression of the activated form of Notch1 (NICD1) promotes ICC development,^20, 22^ and accelerates thioacetamide-induced cholangiocarcinogenesis in mice.^23^

Numerous studies have focused on the role of Notch receptors in ICC development.^20, 22^ However, how these receptors are activated along cholangiocarcinogenesis remains obscure. Since Notch receptor genes are not mutated in ICC, it is plausible to hypothesize that specific upstream signals are needed to activate Notch receptors in this disease. Among Notch ligands, Jag1 is a well-characterized main regulator of liver development and regeneration.^24^ Jag1 has been implicated in the development of multiple tumour types.^24, 25^ For instance, Jag1 expression has been found to be upregulated and its levels inversely associated with patient's survival in breast, colon and cervical cancers.^26, 27, 28^ Also, silencing of Jag1 in human prostate cancer cells decreases cell invasion and *in vivo* tumour growth.^29^ However, the functional contribution of Jag1 in ICC development has not been studied to date.

In the present study, we hypothesized that Jag1 is a key Notch ligand in intrahepatic cholangiocarcinogenesis. We show that Jag1 is overexpressed in human ICC specimens and regulates the *in vitro* growth of human ICC cell lines via the Notch signalling. *In vivo*, we found that while overexpression of *Jag1* alone is insufficient to promote liver tumour development, Jag1 cooperates with activated AKT to induce ICC formation. Altogether, these data support the critical role of Jag1 in the molecular pathogenesis of ICC.

## Results

### Jag1 expression is upregulated in human ICC samples

**Figure 1:**
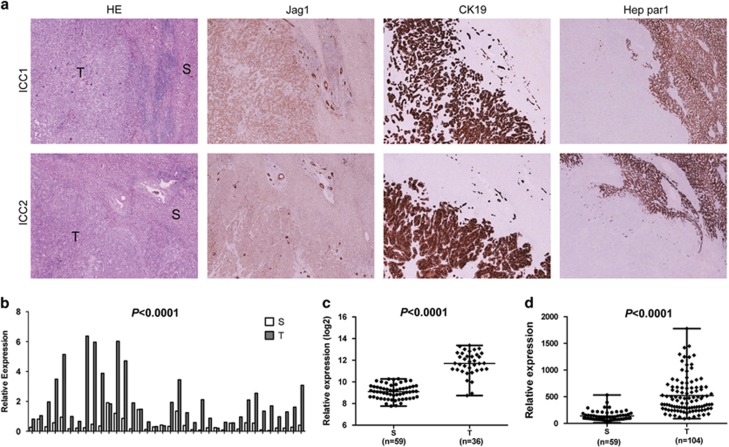
Expression patterns of Jagged 1 (Jag1) in human intrahepatic cholangiocarcinoma (ICC). (**a**) Immunohistochemical patterns of Jag1. Upper panel, ICC case (ICC1) showing upregulation of Jag1 in the tumour part (T), when compared with non-tumorous surrounding liver (S). Lower panel, ICC case (ICC2) showing equivalent levels of Jag1 immunoreactivity in ICC and adjacent non-tumorous liver. Original magnification: × 40. HE, hematoxylin and eosin staining. Cytokeratin 19 (CK19) and Hep par1 were used as biliary and hepatocellular markers, respectively. (**b**) qRT-PCR analysis of *Jag1* mRNA expression in 36 paired human ICC and non-tumour liver tissues (S). The ΔCt value of each sample was calculated by subtracting the average Ct value of the Jag1 gene from the average Ct value of the β-Actin. Tukey−Kramer's test: ****P<0.001. (**c**) Expression of Jag1 in surrounding non-tumour tissues (S) and ICC (T) samples using microarray data retrieved from The Cancer Genome Atlas (TCGA). (**d**) Expression of Jag1 in surrounding non-tumour tissues (S) and ICC (T) samples using microarray data retrieved from Andersen *et al.*^30^

First, we determined the levels of Jag1 in human ICC samples. For this purpose, we examined the protein expression patterns of Jag1 in a collection of human ICC specimens by immunohistochemistry. We found that Jag1 levels are upregulated, when compared with non-tumorous surrounding counterparts, in 81 of 90 (90%) ICC specimens (Figure 1a, upper panel). Equivalent levels of Jag1 immunoreactivity in ICC and corresponding non-neoplastic livers were detected in the remaining samples (Figure 1a, lower panel). No association between the levels of Jag1 and clinicopathologic features of the patients, including age, gender, aetiology, presence of cirrhosis, tumour size, tumour differentiation, tumour number and lymph node metastases, was detected (data not shown).

To further validate the immunohistochemical findings, *Jag1* mRNA levels were analysed in 36 paired human non-tumour liver tissue and ICC specimens from the same collection using real-time quantitative RT-PCR. In accordance with the immunohistochemical data, we found that *Jag1* mRNA expression is significantly upregulated in ICC samples compared to respective non-tumour liver samples (*P*<0.0001; Figure 1b). In particular, higher levels of *Jag1* were detected in 31 of 36 ICC specimens (86.1%) when compared with corresponding non-tumorous surrounding livers. Subsequently, Jag1 mRNA expression was retrieved from The Cancer Genome Atlas (consisting of 36 surgically resected ICCs and 59 surrounding non-tumour liver tissues), as well as the mRNA microarray study by Andersen *et al.*^30^ (consisting of 104 ICC and 59 non-tumour liver tissues). In both datasets, Jag1 mRNA expression was found to be significantly upregulated in human ICC specimens when compared with surrounding non-tumorous livers (*P*<0.0001; Figures 1c and d). Altogether, the present findings indicate that Jag1 expression is almost ubiquitously upregulated in human ICC samples.

### Suppression of Jag1 reduces the *in vitro* growth of human ICC cell lines

**Figure 2:**
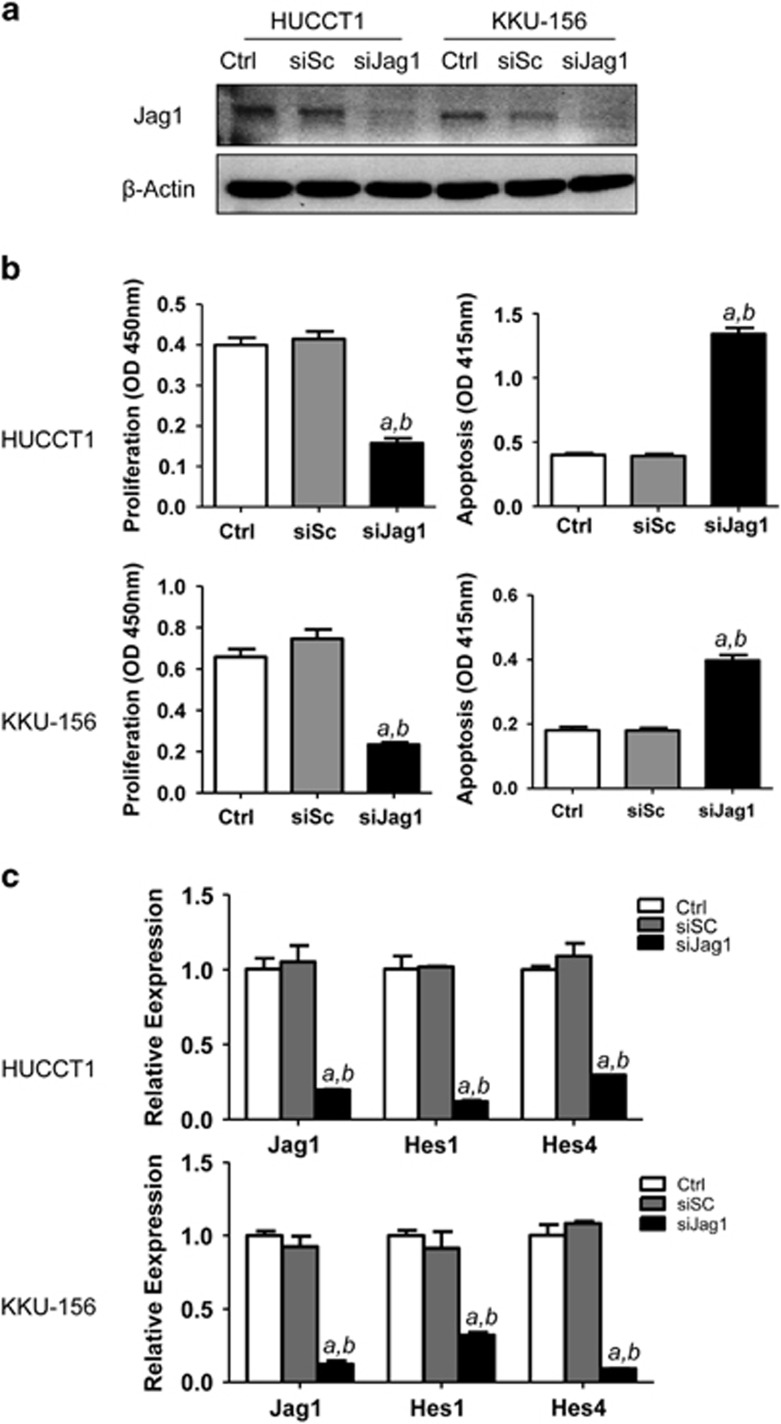
Inhibition of Jag1 restrains the *in vitro* growth of human intrahepatic cholangiocarcinoma (ICC) cell lines. (**a**) Western blotting showing the silencing of Jag1 in HUCCT1 and KKU156 cells. (**b**) Silencing of Jag1 inhibited HUCCT1 and KKU156 cell growth (left panels) and induced apoptosis (right panels); (**c**) Silencing of Jag1 inhibited expression of Notch target gene Hes1 and Hes4 assayed using qRT-PCR. Ctrl: control untreated cells; siSC: scramble siRNA; siJag1: Jag1 siRNA. Tukey–Kramer's test: *P*<0.01 a, vs control; b, vs scramble siRNA.

Subsequently, we assessed the importance of Jag1 on the *in vitro* growth of human ICC cell lines. For this purpose, the *Jag1* gene was silenced using specific small interfering RNA (siRNA) in HUCCT1 and KKU-156 ICC cell lines (Figure 2a). Importantly, knockdown of Jag1 resulted in reduction of proliferation and increase of apoptosis in both cell lines, implying a role for Jag1 in the growth and survival of ICC cells (Figure 2b). At the molecular level, inhibition of Jag1 by siRNA led, as expected, to the downregulation of Notch canonical targets, such as Hes1 and Hes4 (Figure 2c). The present data indicate that inhibition of Jag1 is harmful for the *in vitro* growth of ICC cells, and Jag1 is a major regulator of Notch signalling in human ICCs.

### Jag1 synergizes with activated AKT signalling to promote ICC development in mice

In a previous study, we showed that myristylated/activated AKT1 (myr-AKT1) synergizes with the activated form of Notch1 (NICD1) to induce ICC development in mice.^22^ Thus, we hypothesized that Jag1 overexpression, due to its role as a Notch ligand, might promote ICC development in combination with activated AKT. To test this hypothesis, we hydrodynamically injected the *Jag1* gene, either alone or in combination with *myr-AKT1* (that will be referred to as AKT/Jag1), into the mouse liver. In accordance with previous findings,^31^ overexpression of *myr-AKT1* (*n*=6) led to hepatic steatosis and, eventually, the development of multiple hepatocellular carcinomas (HCC) in each mouse by 28 weeks post-injection (data not shown). Of note, only one ICC in one of six myr-AKT1 mice was detected (data not shown). Overexpression of *Jag1* alone did not lead to any liver abnormality, even 36 weeks post hydrodynamic injection (*n*=8). Histological examination showed that liver parenchyma of Jag1 mice is completely normal (Supplementary Figure 1), indistinguishable from that of wild-type mice, either uninjected (*n*=5) or injected with empty vector (*n*=5).

**Figure 3:**
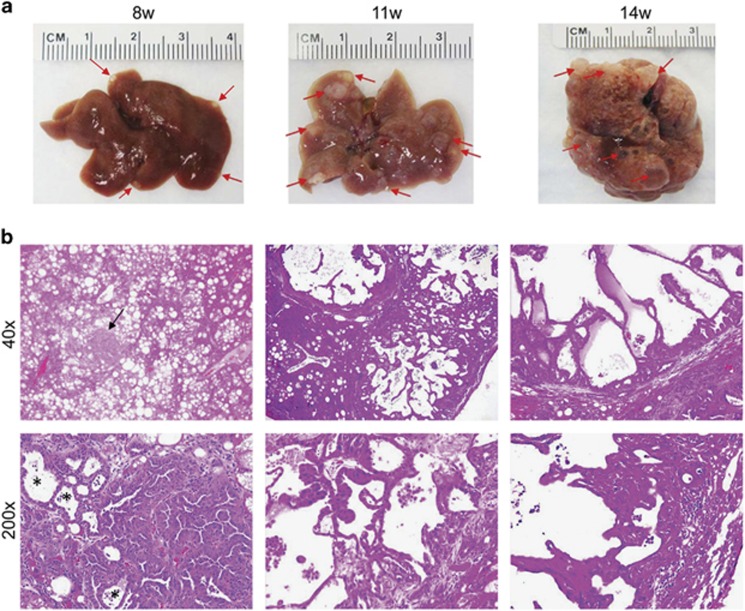
Development of ICC in AKT/Jag1 mice. (**a**) Left panel: 8 weeks post-injection the liver of AKT/Jag1 mice shows the presence of small cystic lesions (indicated by arrows) appearing paler than the surrounding liver parenchyma. These lesions increase in size and number 11 weeks post injection (middle panel), occupying the whole liver by 14 weeks post injection (right panel). (**b**) Left panel: at microscopic level the liver of AKT/Jag1 mice is occupied by numerous lipid-rich preneoplastic lesions equivalent to those detected in AKT mice. However, some cholangiocellular lesions (indicated by arrows) start to emerge. At higher magnification, these lesions are characterized by the presence of solid parts and pseudoglandular formations (the latter indicated by asterisks). With time these lesions occupy the liver parenchyma and tend to form large cysts (middle and right panel). Original magnifications in **b**: × 40 and × 200.

When *myr-AKT1* and *Jag1* were co-injected into the mice, lipid-rich hepatocytes characteristic of myr-AKT1 mice formed small cell clusters starting 3 weeks post injection. Notably, lipid-rich cells were surrounded by small duct-like cells that were positive for the CK19 biliary marker in AKT/Jag1 mice, whereas the duct-like cells were absent in the livers of myr-AKT1 mice (Supplementary Figure 2). Macroscopically, cystic lesions were observed on the liver surface of AKT/Jag1 mice as early as 8 weeks post injection (Figure 3a, left panel). Tumour lesions grew rapidly, and multiple large nodules were visible macroscopically on AKT/Jag1 liver parenchyma 11 weeks post injection (Figure 3a, middle panel). Fourteen weeks post injection, all mice exhibited high tumour burden and deteriorated, being required to be euthanized (Figure 3a, right panel).

**Figure 4:**
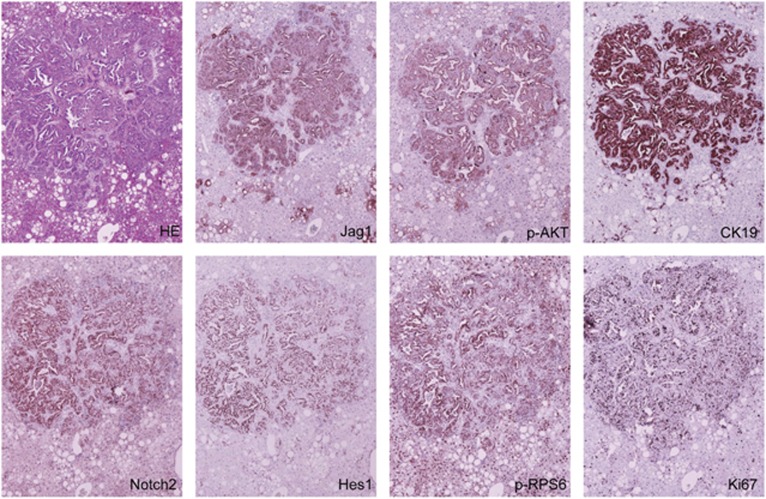
Representative immunohistochemical patterns of an ICC developed in AKT/Jag1 mice. The tumour exhibits homogeneous immunoreactivity for Jag1 and activated/phosphorylated(p)-AKT, thus implying its origin from doubly transfected cells. CK19 staining is used as a biliary marker, ubiquitously positive in ICC lesions. In addition, the cholangiocellular lesion shows intense immunolabelling for markers of the Notch (Notch2, Hes1) and mTOR (phosphorylated/activated RPS6) pathways. The lesion is highly proliferating, as demonstrated by strong immunoreactivity for Ki67 staining. Original magnification: × 100.

**Figure 5:**
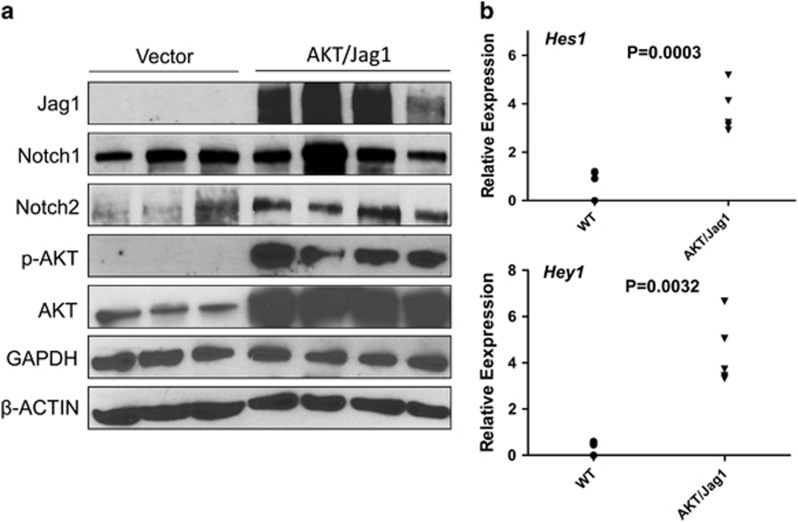
Expression patterns of Notch and AKT pathways in AKT/Jag1 mice. (**a**) Representative western blot analysis showing induction of the Notch (Notch2) and AKT (activated/phosphorylated AKT) pathways in the lesions of AKT/Jag1 mice (14 weeks post injection) when compared with livers injected with the empty vector (vector). (**b**) Quantitative real-time RT-PCR data showing upregulation of the Notch downstream effectors Hes1 and Hey1 in AKT/Jag1 lesions.

Histologically, the tumours exhibited a solid, ductular or cystic phenotype resembling human ICC (Figure 3b). Consistent with histopathological analysis, all tumour cells express ICC specific markers, CK7 (not shown) and CK19 (Figure 4). In addition, immunohistochemical staining showed that AKT/Jag1 cholangiocellular lesions were uniformly positive for phosphorylated/activated (p-)AKT and Jag1 as well as for p-RPS6, a downstream effector of mTOR, and members of the Notch pathway (Notch2 and Hes1; Figure 4), with Notch1 not being induced (data not shown). The cholangiocellular lesions were highly proliferative, as indicated by Ki67 staining. The immunohistochemical data were confirmed by western blotting (Figure 5a) and qRT-PCR (Figure 5b). It is important to note that overexpression of Jag1 alone does not lead to increased Notch2 or p-AKT levels in the mouse liver (Supplementary Figure 3). Also, in accordance with the notion that desmoplastic reaction is a prominent feature of human ICC,^32, 33^ AKT/Jag1 tumours displayed elevated immunoreactivity for smooth muscle actin (α-SMA) and vimentin (VIM) staining in the stromal cells as well as deposition of collagen fibres, as indicated by Picro Sirius Red staining (Supplementary Figure 4).

Altogether, the present results indicate that overexpression of Jag1 cooperates with activated AKT to induce ICC development in the mouse liver.

### Active Notch pathway is required for cholangiocarcinogenesis in AKT/Jag1 mice

**Figure 6:**
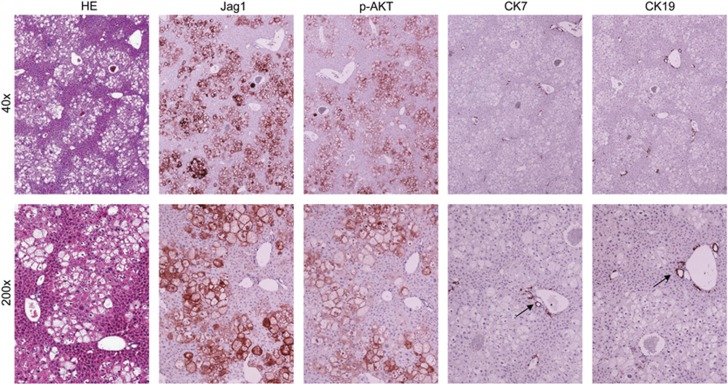
Inhibition of the Notch pathway suppresses intrahepatic cholangiocarcinoma development in AKT/Jag1 mice. Co-injection of AKT/Jag1 with a dominant negative form of the Notch transcriptional activator RBPJ inhibits ICC development in AKT/Jag1 mice. Livers of these mice are characterized by the presence of lipid-rich, hepatocellular lesions identical to those detected in AKT mice, while no cholangiocellular lesions occur. As a consequence, immunoreactivity for biliary markers, such as CK7 and CK19, is limited to normal biliary cells (indicated by arrows). Original magnifications: × 40 and × 200.

Our molecular analysis demonstrated the upregulation of Notch target genes including Notch2, Hes1 and Hey1 by immunostaining (Figure 4) and/or qRT-PCR (Figure 5b), thus supporting the activation of canonical Notch signalling in AKT/Jag1 ICC lesions. Next, we determined whether an active Notch pathway is necessary for ICC development in AKT/Jag1 mice. For this purpose, *myr-AKT1* and *Jag1* were co-injected with a dominant negative form of the Notch transcriptional activator RBP-J (*dnRBP-J*; these mice will be referred to as AKT/Jag1/dnRBP-J). It has been shown that dnRBP-J suppresses the canonical Notch pathway both *in vitro* and *in vivo*.^34^ Of note, combined injection of *myr-AKT1/Jag1* and dn*RBP-J* resulted in the complete abrogation of ICC development 32 weeks post injection (*n*=5; Figure 6). Indeed, at this time point, AKT/Jag1/dnRBP-J mice showed only the presence of lipid-rich preneoplastic lesions, which were identical to those detected in mice injected solely with *myr-AKT1*.^31^ Thus, the present data indicate that Jag1 promotes the development of ICC in AKT/Jag1 mice through the canonical Notch cascade.

## Discussion

Mounting evidence underlines the critical role of Notch signalling in ICC pathogenesis.^10, 11^ For instance, Notch receptors are upregulated in human ICC specimens,^35^ and high expression of Notch1 is associated with invasive ICC growth.^36^ In addition, treatment of human ICC cells with a γ-secretase inhibitor was found to effectively inhibit ICC cell growth and induce apoptosis *in vitro*.^20, 37^ Also, it has been shown that overexpression of the intracellular domain of Notch1 (NICD1) is sufficient to induce ICC formation in mice.^20, 38^ This process can be significantly accelerated by co-expression of activated AKT.^22^ Furthermore, transgenic overexpression of intracellular domain of Notch2 (NICD2) resulted in biliary hyperplasia, and treatment of these mice with the hepatocarcinogen diethylnitrosamine (DEN) eventually led to ICC development.^39^ Similarly, Notch signalling was found to be required for cholangiocarcinogenesis in a chemically induced model of ICC.^23^ Thus, human studies, *in vitro* cell line experiments and mouse models all strongly support the hypothesis that Notch signalling is a central player along ICC development and progression.

Nonetheless, despite this consistent body of data, important issues concerning the Notch pathway in human cholangiocarcinogenesis remain unresolved. In particular, the molecular mechanisms responsible for activation of the Notch signalling have not been investigated in detail to date. For instance, whole genome sequencing analysis did not identify mutations in members of the Notch cascade,^40, 41^ suggesting that activation of Notch is presumably regulated at transcriptional or post-transcriptional level. One possible mechanism triggering activation of Notch signalling is the upregulation of Notch ligands. In the present manuscript, we clearly show that Notch ligand Jag1 might be an important activator of Notch activation in ICC. This assumption is based on the following body of data: (a) Jag1 is overexpressed in the vast majority of human ICC specimens; (b) modulation of Jag1 levels influences the *in vitro* growth as well as Notch pathway activities of human ICC cells; and (c) overexpression of Jag1 synergizes with activated AKT/mTOR signalling to promote ICC development in mice. Although our investigation provides an explanation for Notch activation in ICC, additional work is necessary on this topic. In particular, it is of high importance to unravel the molecular mechanisms whereby Jag1 is induced in this tumour type. Expression analysis conducted either in our or other laboratories demonstrated that *Jag1* mRNA is strongly upregulated in human ICC tissue samples (Figures 1b–d), thus suggesting that Jag1 is predominantly regulated at the transcriptional level. In our previous studies, we found that Jag1 is a transcriptional target of Yap, the transcriptional co-activator downstream of Hippo tumour suppressor pathway, in multiple tumour types.^42^ We also showed that Yap is frequently activated in human ICC cells.^43^ However, our preliminary data seem to exclude that Jag1 is a Yap target in human ICC (Pilo *et al.* unpublished observation). Interestingly, some studies have shown that Jag1 promoter is hypomethylated in breast cancer cells.^44^ Also, Jag1 promoter has been found to be a target of histone demethylase KDM4C.^45^ Thus, additional studies are necessary to elucidate whether the aforementioned epigenetic modifications may also regulate Jag1 expression in human ICCs.

Another important achievement of the present study is the establishment of a novel murine ICC model by hydrodynamic transfection of *myr-AKT1* and *Jag1*. AKT/Jag1-induced ICC tumours exhibited increased cell proliferation and extensive stromal reaction as well as high levels of AKT/mTOR and Notch activation. All these histological and biochemical features are consistent with what has been described in human ICC. Therefore, the AKT/Jag1 mouse represents a useful model to further investigate the molecular mechanisms responsible for ICC initiation and progression. When compared with the AKT/NICD model that we have previously established,^22^ the AKT/Jag1 mouse is more suitable for experimental therapeutics as it can be used to evaluate the potential of Notch inhibitors for the treatment of ICC. Indeed, the AKT/NICD mouse overexpresses the intracellular domain of Notch1 and, therefore, cannot be used to test Notch inhibitors, including γ-secretase inhibitors, anti-Notch or anti-Jag1 antibodies. In a recent study from our lab, we showed that anti-Notch2 and anti-Jag1 antibodies were able to inhibit the growth of ICC-like lesions in AKT/Ras mice.^46^ The study suggests that anti-Notch2 and anti-Jag1 antibodies might represent novel and effective treatment for ICC. However, as AKT/Ras co-expression in the liver induces the occurrence of HCC, ICC and mixed HCC/ICC,^47^ the precise therapeutic potential of these anti-Notch antibodies cannot be accurately evaluated in this mouse model. Thus, the AKT/Jag1 model might represent an excellent murine model to establish whether anti-Notch2 or anti-Jag1 antibodies can be used for the treatment of this deadly disease.

## Materials and methods

### Human tissue samples

A collection of formalin-fixed, paraffin-embedded ICC (*n*=90) samples was used in the present study. Thirty-six frozen ICC and corresponding non-tumorous surrounding livers from the same collection were also used. The clinicopathological features of liver cancer patients are summarized in Supplementary Table 1. ICC specimens were collected at the Medical Universities of Greifswald (Greifswald, Germany) and Sassari (Sassari, Italy). Institutional Review Board approval was obtained at the local Ethical Committee of the Medical Universities of Greifswald and Sassari. Informed consent was obtained from all subjects.

### Constructs and reagents

The constructs used in the experiments, including pT3-EF1α-myr-AKT, pT3-EF1α-Jag1 and pCMV/sleeping beauty transposase (SB), have been previously described.^42, 48, 49^ To generate a dominant negative form of RBP-J (dnRBP-J), the mutant and dominant negative form of RBP-J (RBP-J(R218H),^34^ kindly provided by Dr Tasuku Honjo (Kyoto University, Japan), was cloned in pT3-EF1α vector via Gateway cloning strategy. Plasmids were purified using the Endotoxin free Maxi-Prep Kit (Sigma-Aldrich, St Louis, MO, USA) before being injected into mice.

### Hydrodynamic injection, mouse monitoring

FVB/N mice were purchased from the Jackson Laboratory (Bar Harbor, ME, USA) and they were randomly selected for the animal experiments described in this manuscript. Both male and female mice were used. Hydrodynamic injection was performed as described previously.^50^ In brief, 20 μg pT3-EF1α-myr-AKT1 and 20 μg pT3-EF5α-Jagged1 along with sleeping beauty transposase (SB) in a ratio of 25:1 were diluted in 2 ml saline (0.9% NaCl), then filtered through 0.22 μm filter, and injected into the lateral tail vein of 6 to 8-week-old *FVB* mice in 5–7 seconds. To block the Notch cascade, high doses of dnRBP-J (20 μg) with low doses of myr-AKT1 (4 μg) and Jag1 (4 μg) were injected. Investigators were not blinded for the animal studies. At least five mice were used in each cohort in each experiment; and the number of mice was determined according to prior experience of *in vivo* studies in our laboratory. All animals with successful hydrodynamic injection were included for data analysis. The care and use of mice for this study were carried out with the approval of the Institutional Animal Care and Use Committee (IACUC) of the University of California, San Francisco, USA.

### Histology and immunohistochemistry

Liver lesions were fixed in 4% paraformaldehyde overnight at 4 °C and embedded in paraffin by two board-certified pathologists (SR and FD) in accordance with the criteria by Frith *et al.*^51^ For immunohistochemistry, antigen retrieval was performed in 10 mM sodium citrate buffer (pH 6.0) by placement in a microwave on high for 10 min, followed by a 20-min cool down at room temperature. After a blocking step with the 5% goat serum and Avidin-Biotin blocking kit (Vector Laboratories, Burlingame, CA, USA), the slides were incubated with primary antibodies overnight at 4 °C. Slides were then subjected to 3% hydrogen peroxide for 10 min to quench endogenous peroxidase activity and subsequently the biotin conjugated secondary antibody was applied at a 1:500 dilution for 30 min at room temperature. Anti-phosphorylated/activated-Akt (3787), Vimentin (5741) and HA tag (3724) antibodies were obtained from Cell Signaling Technology Inc (Danvers, MA, USA); anti-Jag1 (ab109536), anti-CK7 (ab181598) and anti-CK19 (ab133496) antibodies were obtained from Abcam (Cambridge, MA, USA); anti-α smooth muscle actin (SMA; M0851) antibody was obtained from Dako Cytomation (Carpinteria, CA, USA); and KI67 antibody (IHC-00375) was purchased from Bethyl Laboratories (Montgomery, TX, USA). These primary antibodies were selected since they have been extensively validated by the manufacturers for immunohistochemistry. The immunoreactivity was visualized with the Vectastain Elite ABC kit (Vector Laboratories), using Vector NovaRED™ (Vector Laboratories) as the chromogen. Slides were counterstained with Mayer's hematoxylin. Picro Sirius Red staining was performed using standard methods. Immunohistochemical expression of the Jag1 protein was evaluated semi-quantitatively by direct comparison of the tumour area (cholangiocarcinoma) with the adjacent non-neoplastic liver parenchyma. Specifically, the Jag1 staining scores were determined as follows: (a) equivalent, when the immunoreactivity was similar between cholangiocarcinoma tissues and corresponding surrounding non-tumorous liver tissues; (b) overexpression, when immunoreactivity intensity was higher in cholangiocarcinoma tissues than in corresponding surrounding non-tumorous liver tissues.

### Western blotting

Mouse livers tissues were homogenized in lysis buffer [30 mM Tris (pH 7.5), 150 mM NaCl, 1% NP-40, 0.5% Na deoxycholate, 0.1% SDS, 10% glycerol and 2 mM EDTA] containing the Complete Protease Inhibitor Cocktail (ThermoFisher Scientific, Waltham, MA, USA). Protein concentrations were determined with the Bio-Rad Protein Assay Kit (Bio-Rad, Hercules, CA, USA) using bovine serum albumin as standard. For western blotting, aliquots of 40 μg were denatured by boiling in Tris-Glycine SDS Sample Buffer (Bio-Rad), separated by SDS-PAGE, and transferred onto nitrocellulose membranes (Bio-Rad) by electroblotting. Membranes were blocked in5% non-fat dry milk in Tris-buffered saline containing 0.1% Tween-20 for 1 h and probed with following specific antibodies for Notch2 (5732), Notch1 (3608), p-AKT (3787), total-AKT (9272) (Cell Signaling Technology Inc), and Jagged1 (Abcam, ab109536). Anti-GAPDH (MAB374, EMD Millipore, Billerica, MA, USA) and anti-β-actin (A5441, Sigma-Aldrich) were used as loading controls. Each primary antibody was followed by incubation with horseradish peroxidase-secondary antibody (Jackson ImmunoResearch Laboratories Inc., West Grove, PA, USA) diluted 1:5000 for 30 min and proteins were revealed with the Super Signal West Dura (ThermoFisher Scientific).

### *In vitro* studies

The HUCCT1 and KKU-156 ICC cell lines were kindly provided to us by Dr Gores at Mayo Clinics. All cells were grown in a 5% CO_2_ atmosphere, at 37 °C, in RPMI Medium supplemented with 10% fetal bovine serum (FBS, ThermoFisher Scientific) and penicillin/streptomycin (ThermoFisher Scientific). Cell proliferation was analysed using the BrdU Cell Proliferation Assay Kit (Cell Signaling Technology Inc.). Apoptosis was assessed with the Cell Death Detection Elisa Plus Kit (Roche Molecular Biochemicals, Indianapolis, IN, USA).

### Knockdown of Jag1

For knockdown studies, HUCCT1 and KKU-156 ICC cells were transfected with 50 nM siRNA targeting *Jag1* (ID # s1175; ThermoFisher Scientific). A scramble siRNA (ID # 4390846; ThermoFisher Scientific) was used as negative control. RNA was extracted 48 h after transfection. Experiments were repeated at least three times in triplicate.

### Quantitative reverse transcription real-time polymerase chain reaction (qRT-PCR)

Validated Gene Expression Assays for human *Jag1* (Hs01070032_m1), *Hes1* (Hs00172878_m1), *Hes4* (Hs00368353_g1) and β-*Actin* (ID: 4333762T) were purchased from ThermoFisher Scientific. PCR reactions were performed with 100 ng of cDNA on the whole sample collection and cell lines, using an ABI Prism 7000 Sequence Detection System and TaqMan Universal PCR Master Mix (ThermoFisher Scientific). Cycling conditions were: 10 min of denaturation at 95 °C and 40 cycles at 95 °C for 15 s and at 60 °C for 1 min. Quantitative values were calculated by using the PE Biosystems Analysis software and expressed as N target (NT). NT=2^−ΔCt^, wherein ΔCt value of each sample was calculated by subtracting the average Ct value of the target gene from the average Ct value of the β-*Actin* gene.

### Statistical analysis

All data were analysed with the Prism 6 software (GraphPad, San Diego, CA, USA). Comparisons between two groups were performed with two-tailed unpaired or paired *t*-test. Statistical differences among the various groups were assessed with the Tukey–Kramer's test. All graphs are the mean ± SEM.
