# Supplementary material for: Jagged 1 is a major Notch ligand along cholangiocarcinoma development in mice and humans
Source: Oncogenesis. 2016 Dec 5;5(12):e274–. doi: 10.1038/oncsis.2016.73 (PMC5177771; doi:10.1038/oncsis.2016.73)
Supplement: Supplementary Table 1 [file oncsis201673x1.doc]

**Supplementary Table 1**. Clinicopathological features of intrahepatic cholangiocarcinoma (ICC) patients

| Variables | | | |
| --- | --- | --- | --- |
| No. of patients  Male  Female | 90  66  24 | |  |
| Age (years)  <60  >60 | 38  52 | |  |
| Etiology  HBV  HCV  Hepatolithiasis  PSC  NA | 14  18  26  5  27 | |  |
| Liver cirrhosis  Yes  No | 25  65 | |  |
| Tumor differentiation  Well  Moderately  Poorly | 34  29  27 | |  |
| Tumor size (cm)  <5 51  >5 39 | |  | |
| Tumor number  Single 58  Multiple 32 | |  | |
| Lymph node metastases  Yes 14  No 46  NA 30 | |  | |

Abbreviations: NA, not available; PSC, primary sclerosing cholangitis
