# Supplementary material for: Jagged 1 is a major Notch ligand along cholangiocarcinoma development in mice and humans
Source: Oncogenesis. 2016 Dec 5;5(12):e274–. doi: 10.1038/oncsis.2016.73 (PMC5177771; doi:10.1038/oncsis.2016.73)
Supplement: Supplementary Figure 1 [file oncsis201673x2.ppt]

## Slide 1
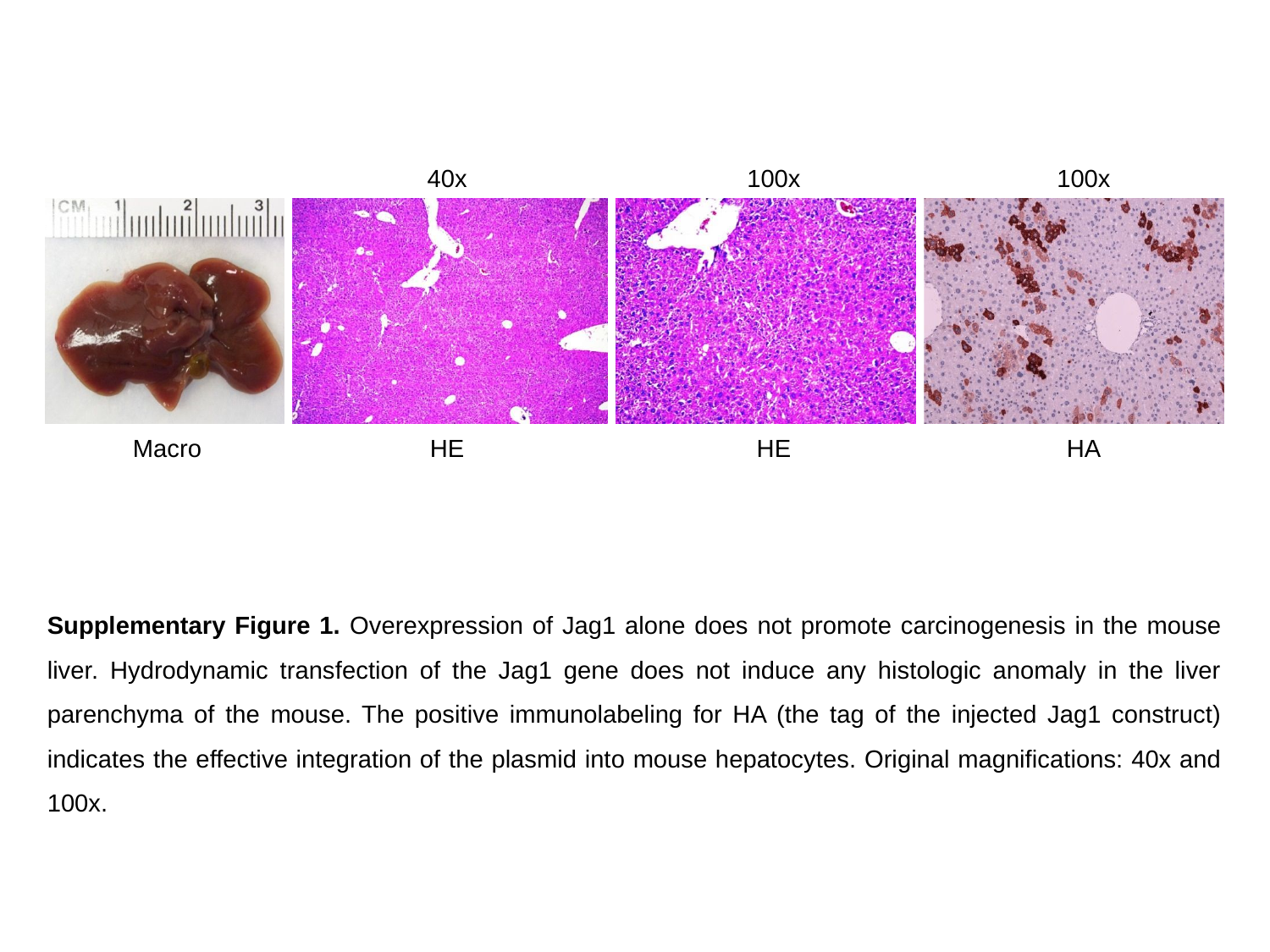

40x
100x
100x
Macro
HE
HE
HA
Supplementary Figure 1. Overexpression of Jag1 alone does not promote carcinogenesis in the mouse liver. Hydrodynamic transfection of the Jag1 gene does not induce any histologic anomaly in the liver parenchyma of the mouse. The positive immunolabeling for HA (the tag of the injected Jag1 construct) indicates the effective integration of the plasmid into mouse hepatocytes. Original magnifications: 40x and 100x.
