# Supplementary material for: Jagged 1 is a major Notch ligand along cholangiocarcinoma development in mice and humans
Source: Oncogenesis. 2016 Dec 5;5(12):e274–. doi: 10.1038/oncsis.2016.73 (PMC5177771; doi:10.1038/oncsis.2016.73)
Supplement: Supplementary Figure 2 [file oncsis201673x3.ppt]

## Slide 1
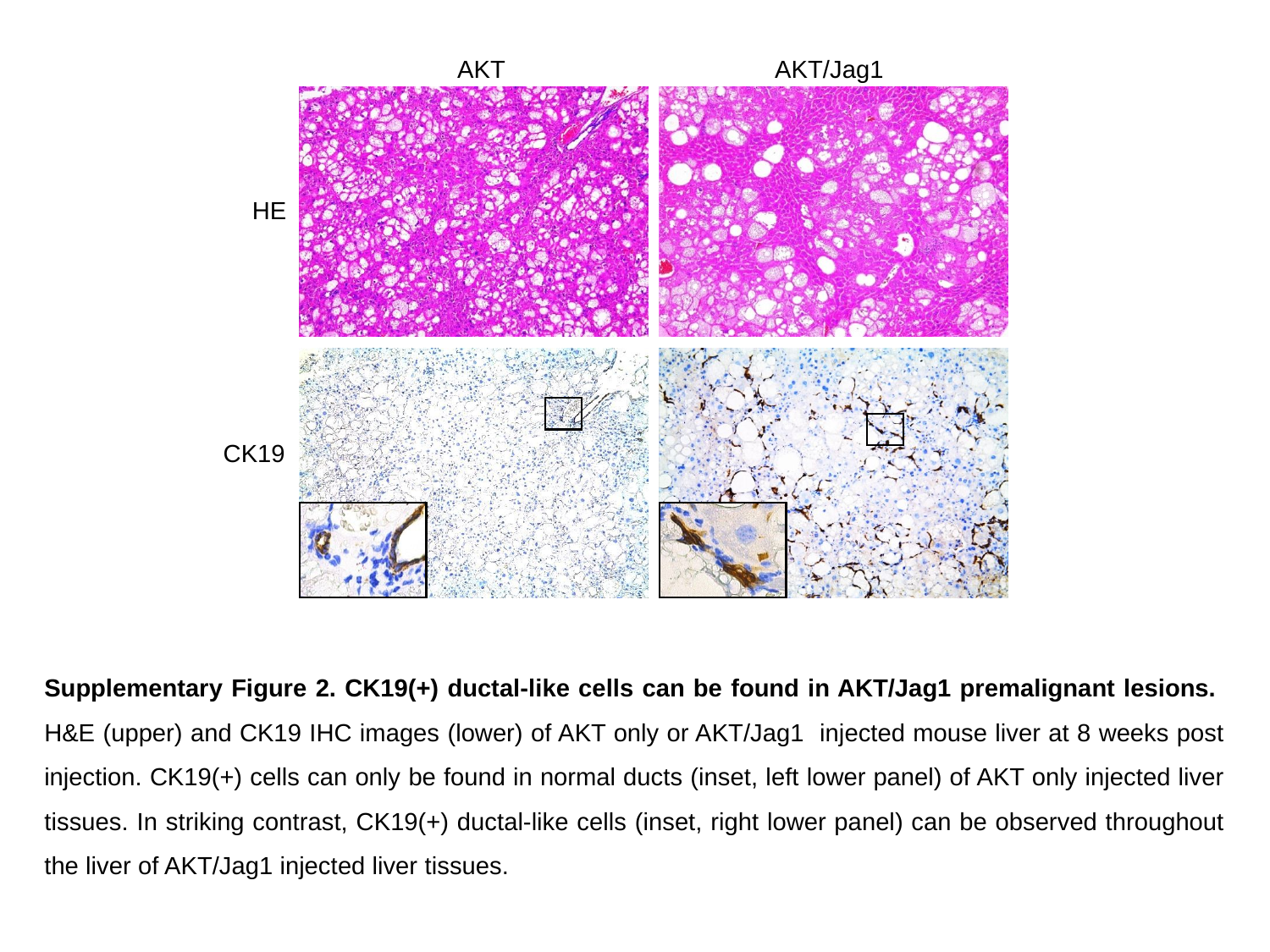

AKT
AKT/Jag1
 HE
CK19
Supplementary Figure 2. CK19(+) ductal-like cells can be found in AKT/Jag1 premalignant lesions. H&E (upper) and CK19 IHC images (lower) of AKT only or AKT/Jag1 injected mouse liver at 8 weeks post injection. CK19(+) cells can only be found in normal ducts (inset, left lower panel) of AKT only injected liver tissues. In striking contrast, CK19(+) ductal-like cells (inset, right lower panel) can be observed throughout the liver of AKT/Jag1 injected liver tissues.
