# Supplementary material for: Jagged 1 is a major Notch ligand along cholangiocarcinoma development in mice and humans
Source: Oncogenesis. 2016 Dec 5;5(12):e274–. doi: 10.1038/oncsis.2016.73 (PMC5177771; doi:10.1038/oncsis.2016.73)
Supplement: Supplementary Figure 3 [file oncsis201673x4.ppt]

## Slide 1
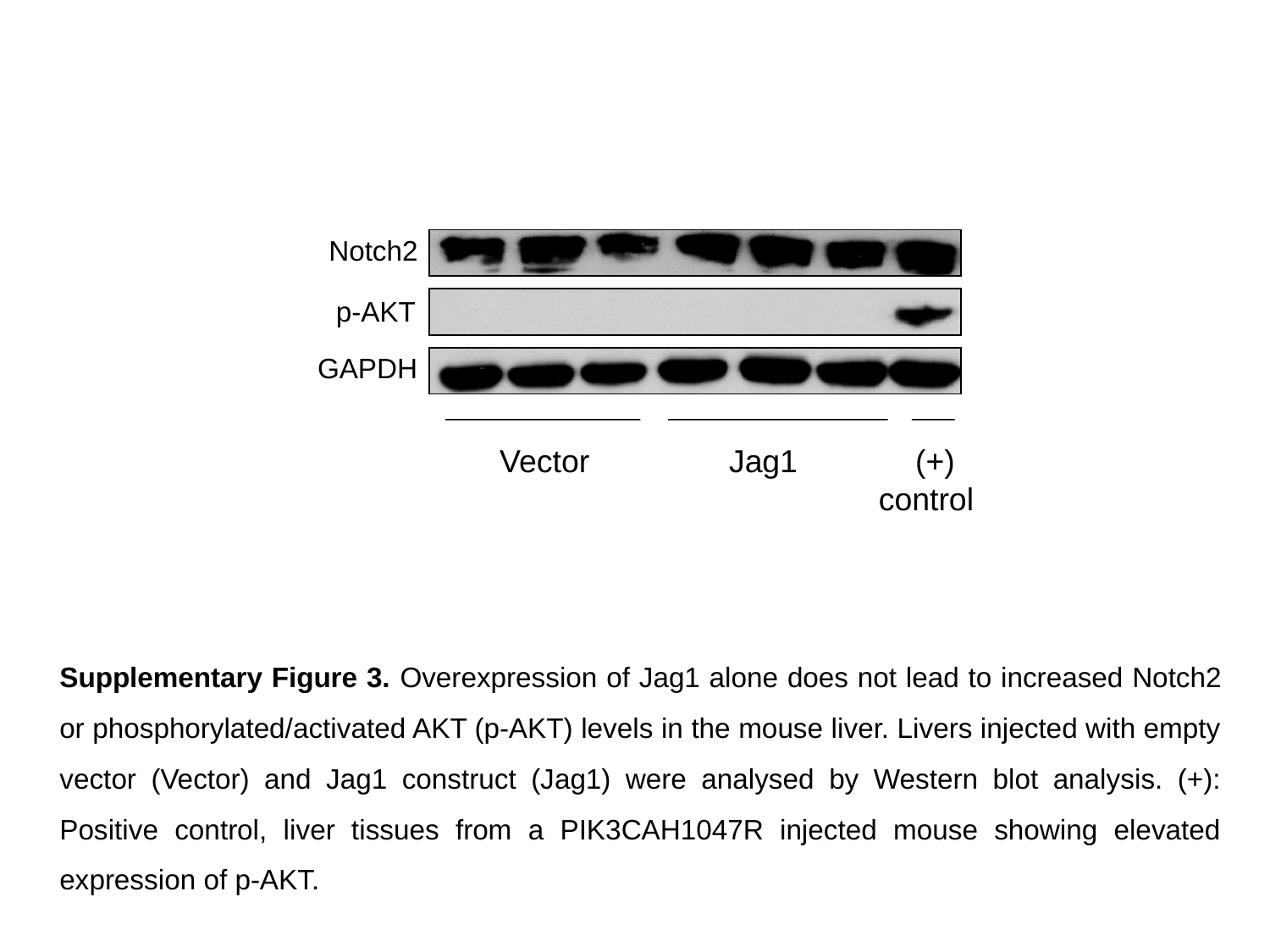

Notch2
p-AKT
GAPDH
Vector
Jag1
 (+) control
Supplementary Figure 3. Overexpression of Jag1 alone does not lead to increased Notch2 or phosphorylated/activated AKT (p-AKT) levels in the mouse liver. Livers injected with empty vector (Vector) and Jag1 construct (Jag1) were analysed by Western blot analysis. (+): Positive control, liver tissues from a PIK3CAH1047R injected mouse showing elevated expression of p-AKT.
