# Supplementary material for: Jagged 1 is a major Notch ligand along cholangiocarcinoma development in mice and humans
Source: Oncogenesis. 2016 Dec 5;5(12):e274–. doi: 10.1038/oncsis.2016.73 (PMC5177771; doi:10.1038/oncsis.2016.73)
Supplement: Supplementary Figure 4 [file oncsis201673x5.ppt]

## Slide 1
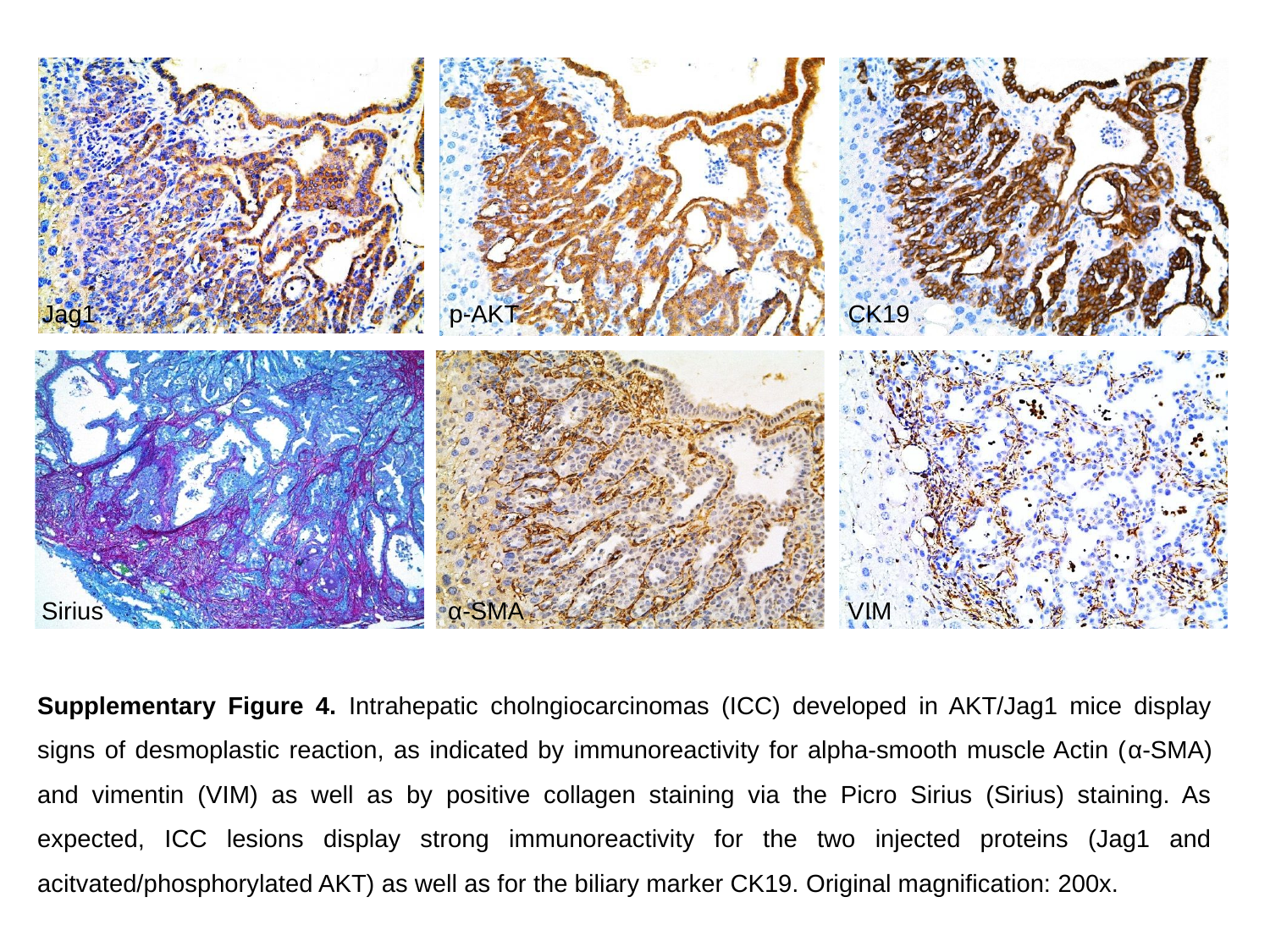

Jag1
p-AKT
CK19
Sirius
α-SMA
VIM
Supplementary Figure 4. Intrahepatic cholngiocarcinomas (ICC) developed in AKT/Jag1 mice display signs of desmoplastic reaction, as indicated by immunoreactivity for alpha-smooth muscle Actin (α-SMA) and vimentin (VIM) as well as by positive collagen staining via the Picro Sirius (Sirius) staining. As expected, ICC lesions display strong immunoreactivity for the two injected proteins (Jag1 and acitvated/phosphorylated AKT) as well as for the biliary marker CK19. Original magnification: 200x.
